# Supplementary material for: A New Gene SCY3 Homologous to Scygonadin Showing Antibacterial Activity and a Potential Role in the Sperm Acrosome Reaction of Scylla paramamosain
Source: Int J Mol Sci. 2023 Mar 16;24(6):5689. doi: 10.3390/ijms24065689 (PMC10053787; doi:10.3390/ijms24065689)
Supplement: Supplementary file 1 [file ijms-24-05689-s001.zip › ijms-2237831-supplementary.pdf]

Supplementary Information

For

A New Gene *SCY3* Homologous to *Scygonadin* Showing Antibacterial Activity and a Potential Role in the Sperm Acrosome Reaction of *Scylla paramamosain*

Su Long [1], Fangyi Chen [1,2,3], Jishan Li [1], Ying Yang [1] and Ke-Jian Wang [1,2,3,*]

[1] State Key Laboratory of Marine Environmental Science, College of Ocean & Earth Sciences, Xiamen University, Xiamen 361102, China
[2] State-Province Joint Engineering Laboratory of Marine Bioproducts and Technology, College of Ocean & Earth Sciences, Xiamen University, Xiamen 361102, China
[3] Fujian Innovation Research Institute for Marine Biological Antimicrobial Peptide Industrial Technology, College of Ocean & Earth Sciences, Xiamen University, Xiamen 361102, China
* Correspondence: wkjian@xmu.edu.cn

Figures and Tables

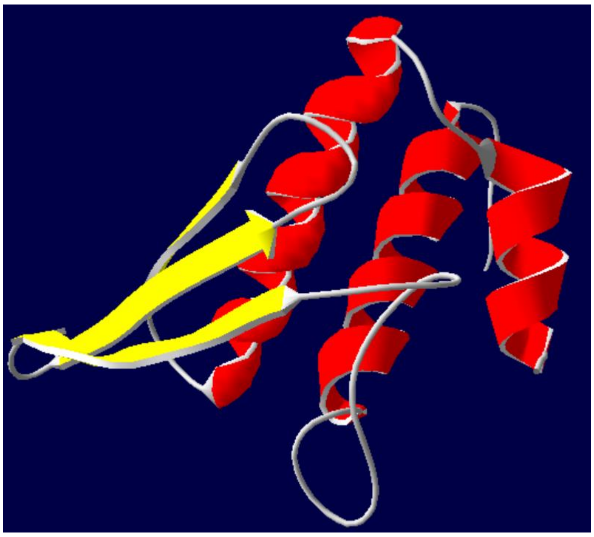

Figure S1. Prediction of the tertiary structure of SCY3 by PHYRE2.

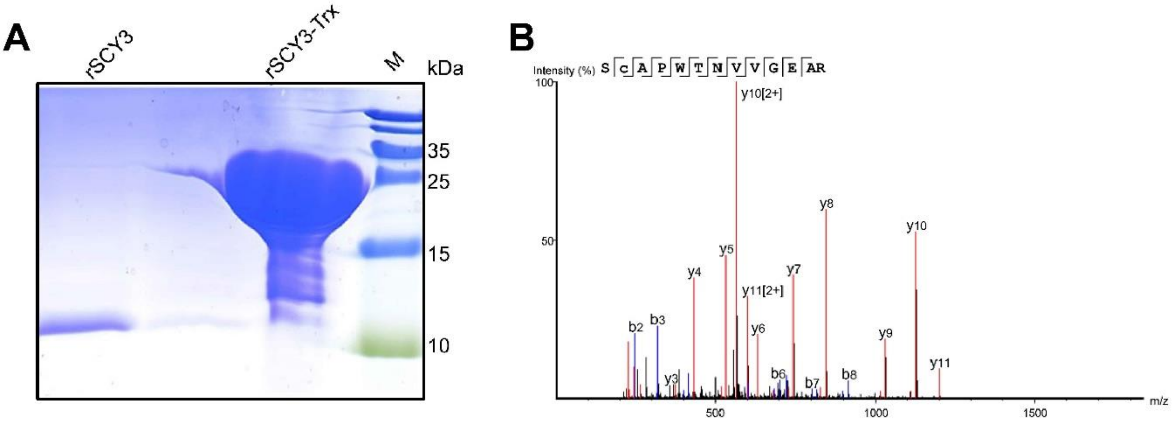

Figure S2. The expression and purification of SCY3 in eukaryotic expression system and prokaryotic expression system, and the mass spectrometry result. (A) SDS-PAGE analysis of purification results of rSCY3 in eukaryotic expression system and prokaryotic expression system. M, marker; 1, purified protein rSCY3 in eukaryotic expression system; 2, purified protein rSCY3 in prokaryotic expression system. (B) The mass spectrometry result of eukaryotic expression product rSCY3.

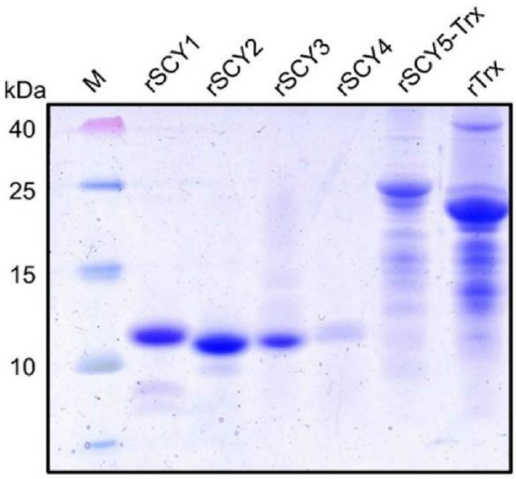

Figure S3. The expression and purification of rSCY1, rSCY2, rSCY3, rSCY4 in eukaryotic expression system and rSCY5, rTrx in prokaryotic expression system. M, marker; 1, purified

protein rSCY1 in eukaryotic expression system; 2, purified protein rSCY2 in eukaryotic expression system; 3, purified protein rSCY3 in eukaryotic expression system; 4, purified protein rSCY4 in eukaryotic expression system; 5, purified protein rSCY5 in prokaryotic expression system; 6, purified protein rTrx in prokaryotic expression system.

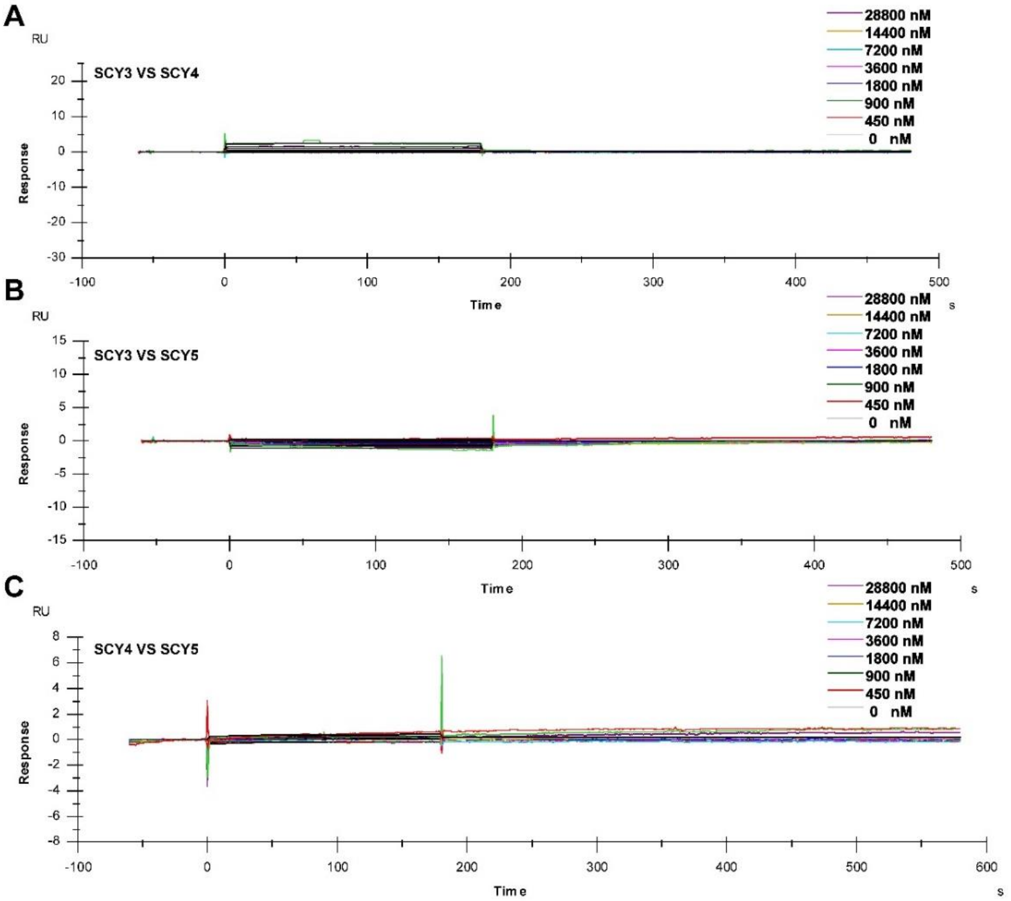

Figure S4. Binding kinetic between rSCY3 and rSCY4 (A), rSCY3 and rSCY5 (B), rSCY4 and rSCY5 assessed by surface plasmon resonance technology.

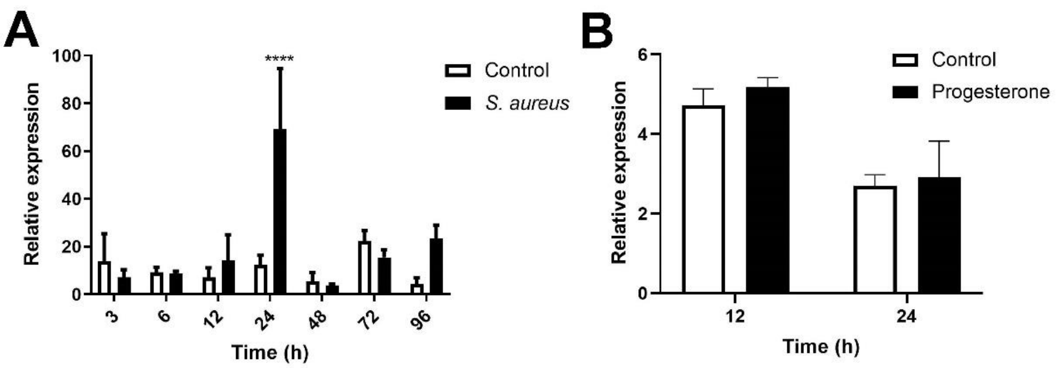

Figure S5. The expression pattern of SCY3 in hemocyte (A) after challenge with *S. aureus* and (B) in *ejaculation duct* stimulated with progesterone. The asterisks indicated a significant difference compared with the control group (****: P < 0.0001).

Table S1: Microbial strains information

Microorganisms	*CGMCC No.a*
Gram-negative bacteria	
Pseudomonas fluorescens	1.0032
Pseudomonas stutzeri	1.1803
Shigella flexneri	1.1868
Escherichia coli	1.2389
Aeromonas hydrophila	1.2017
Vibrio fluvialis	1.1609
Vibrio harveyi	1.1593
Vibrio alginolyticus	1.1833
Vibrio parahaemolyticus	1.1615
Gram-positive bacteria	
Listeria monocytogenes	1.10753
Staphylococcus epidermidis	1.4260
Pseudomonas stutzeri	1.1868
Micrococcus lysodeikticus	1.0634
Micrococcus luteus	1.634
Bacillus subtilis	1.108
Fungi	
Candida albicas	2.2411
Cryptococcus neoformans	2.1563
Fusarium graminearum	3.349
Fusarium solani	3.584
Fusarium oxysporum	3.6785
Aspergillus niger	3.0316
Aspergillus ochraceus	3.583
Aspergillus fumigatus	3.5835

[a]China general microbiological culture collection number.

Table S2: Primers used in the present study.

		SCY3 (5′-3′)
**For RACE PCR**	3-3R1	CCCTCAACAAGCTTCTCCCT
	3-3R2	AGATGTCGTGTGCTCCTTGG
	3-5R1	AGCCAGTCCGTGACTTCTTC
	3-5R2	TGCAGTGTAGGAGCCGTTTT
	UPM-Long	CTAATACGACTCACTATAGGGCAAGCAGTGGTATCAACGCAGAGT
	UPM-short	CTAATACGACTCACTATAGGGC
**For Introns PCR**	3geneF	ATGCGTCCATCTCTCTTGGTCAGTC
	3geneR	TTAGTAGGAAGCTAGCCAGTCCGTG
**For RT-PCR**	3-DL-F	TGAACTCCAGGCAACTAA
	3-DL-R	TCCGTGACTTCTTCTATGG
	GAPDH-F	CTCCACTGGTGCCGCTAAGGCTGTA
	GAPDH-R	CAAGTCAGGTCAACCACGGACACAT
**For recombinant expression**	3-ZH-F	GGAATTCGGCTCCACCCTCAACAAGCTTCTCC
	3-ZH-R	ATAAGAATGCGGCCGCTTAATGGTGATGGTGATGATGGTAGGAAGCTAGCCAGTCCGTG
	3-YH-F	GGAATTCGGCTCCACCCTCAACAAGCTTCTCC
	3-YH-R	ATAAGAATGCGGCCGCTTAGTAGGAAGCTAGCCAGTCCGTG
	T7	TAATACGACTCACTATAGGG
	T7TER	TGCTAGTTATTGCTCAGCGG
	5'AOX	GACTGGTTCCAATTGACAAGC
	3'AOX	GGCAAATGGCATTCTGACAT

The underlined sequences represent restriction sites, the double underscore represents His purification label.

Table S3: Antibacterial activity of rSCY3.

Microorganisms	CGMCC No.[a]	*E. coli*-derived SCY3		*P. pastoris*-derived SCY3		rTrx	
		[b]MIC	[b]MBC	MIC	MBC	MIC	MBC
**Gram-negative bacteria**							
Pseudomonas fluorescens	1.0032	>48	>48	>48	>48	>48	>48
Pseudomonas stutzeri	1.1803	>48	>48	>48	>48	>48	>48
Shigella flexneri	1.1868	>48	>48	>48	>48	>48	>48
Escherichia coli	1.2389	>48	>48	>48	>48	>48	>48
Aeromonas hydrophila	1.2017	>48	>48	>48	>48	>48	>48
Vibrio fluvialis	1.1609	>48	>48	>48	>48	>48	>48
Vibrio harveyi	1.1593	>48	>48	>48	>48	>48	>48
Vibrio alginolyticus	1.1833	>48	>48	>48	>48	>48	>48
Vibrio parahaemolyticus	1.1615	>48	>48	>48	>48	>48	>48
**Gram-positive bacteria**							
Listeria monocytogenes	1.10753	>48	>48	>48	>48	>48	>48
Staphylococcus epidermidis	1.4260	>48	>48	>48	>48	>48	>48
Pseudomonas stutzeri	1.1868	>48	>48	>48	>48	>48	>48
Micrococcocus lysodeikticus	1.0634	>48	>48	>48	>48	>48	>48
Micrococcus luteus	1.634	24-48	24-48	24-48	24-48	24-48	24-48
Bacillus subtilis	1.108	>48	>48	>48	>48	>48	>48
**Fungi**							
Candida albicas	2.2411	>48	>48	>48	>48	>48	>48
Cryptococcus neoformans	2.1563	>48	>48	>48	>48	>48	>48
Fusarium graminearum	3.349	>48	>48	>48	>48	>48	>48
Fusarium solani	3.584	>48	>48	>48	>48	>48	>48
Fusarium oxysporum	3.6785	>48	>48	>48	>48	>48	>48
Aspergillus niger	3.0316	>48	>48	>48	>48	>48	>48
Aspergillus ochraceus	3.583	>48	>48	>48	>48	>48	>48
Aspergillus fumigatus	3.5835	>48	>48	>48	>48	>48	>48

[a]China general microbiological culture collection number; [b]MIC and [b]MBC: All the concentrations showed in this table were in μM. The values of MIC (minimal inhibitory concentration) and MBC (minimal bactericidal concentration) were expressed as the lowest concentration yielding no detectable microbial growth or that killed more than 99.9 % of microorganism.
